# A new side-effect of sufentanil: increased monocyte-endothelial adhesion

**DOI:** 10.1186/s12871-021-01487-3

**Published:** 2021-11-03

**Authors:** Dongdong Yuan, Zhaowei Zou, Xianlong Li, Nan Cheng, Na Guo, Guoliang Sun, Dezhao Liu

**Affiliations:** 1 Department of Anesthesiology, The third affiliated hospital of Sun Yat-sen university, Tianhe Road, Guangzhou, Guangdong P. R. China; 2 Department of General Surgery, Zhujiang Hospital, Southern Medical University, Guangzhou, China

**Keywords:** Monocyte-endothelial adherence, Opioids, Side-effect

## Abstract

**Background:**

Opioids have been identified by the World Health Organization to be ‘indispensable for the relief of pain and suffering’. Side-effects, such as nausea, vomiting, postoperative delirium, and effects on breathing, of opioids have been well investigated; however, the influence of opioids on monocyte-endothelial adherence has never been reported. Therefore, we explored the effects of representative opioids, fentanyl, sufentanil, and remifentanil, on monocyte-endothelial adherence and the underlying mechanisms.

**Methods:**

We built a cell adhesion model with U937 monocytes and human umbilical vein endothelial cells (HUVECs). Two kinds of connexin43 (Cx43) channel inhibitors, 18-α-GA and Gap 27, were used to alter Cx43 channel function in U937 monocytes and HUVECs, respectively, to determine the effects of Cx43 channels on U937-HUVEC adhesion. Subsequently, the effects of fentanyl, sufentanil and remifentanil on Cx43 channel function and U937-HUVEC adhesion were explored.

**Results:**

When fentanyl, sufentanil and remifentanil acted on monocytes or endothelial cells, their effects on monocyte-endothelial adherence differed. When acting on U937 monocytes, sufentanil significantly increased U937-HUVEC adhesion which was associated with reduced release of ATP from Cx43 channels, while fentanyl and remifentanil did not have these influences. Although sufentanil could also inhibit Cx43 channel function in HUVECs, it had no effect on ATP release from HUVECs or U937-HUVECs adhesion.

**Conclusions:**

We demonstrated that sufentanil application increases monocyte-endothelial adherence which was associated with reduced release of ATP from Cx43 channels in monocytes. This side-effect of sufentanil should be considered seriously by clinicians.

**Supplementary Information:**

The online version contains supplementary material available at 10.1186/s12871-021-01487-3.

## Background

Opioids have been identified by World Health Organization to be ‘indispensable for the relief of pain and suffering’ [1, 2]. Opioids act on opioid receptors to produce morphine-like effects and are commonly used for the control of clinical pain in patients with cancer or undergoing surgery. These patients can experience pain from spending long periods in the supine position and are prone to hemodynamic changes. Under these circumstances, monocytes flowing in blood vessels easily adhere to inflamed or damaged vascular endothelial cells [3]. Monocyte-endothelial adherence plays an important part in the initial stages of inflammatory vascular diseases [4]. Adherent monocytes not only damage the vascular endothelium directly but also cause the release of inflammatory factors and chemoattractants indirectly. This process is continuously self-reinforcing, eventually resulting in vascular damage and deterioration, thrombosis formation, and even development of atherosclerosis. Therefore, we believe that monocyte-endothelial adherence is a pre-requisite for vascular damage [5].

In contemporary society, opioids are not only extensively used for the control of cancer pain [6] but also widely used in anaesthesia and postoperative analgesia for surgical patients [7]. In studies on the side-effects of opioids, researchers pay extensive attention common symptoms such as nausea, vomiting, postoperative delirium, and breathing effects; however, the effect of opioids on monocyte-endothelial adherence has never been reported [8–10].

Connexin43 (Cx43), which belongs to a transmembrane protein family known as connexin, has been reported to be associated with monocyte-endothelial adherence [5, 11]. Cx43 forms gap junction channels that mediate cytosolic signalling molecules movement between neighbouring cells. The gap junctions are composed of two hemichannels, which dock together end-to-end [12]. Hemichannels can exist unopposed in plasma membranes and have various functions, such as ATP release [13]. Furthermore, extracellular ATP can be rapidly metabolized to adenosine (ADO), which has well-known anti-inflammatory effects that decrease monocyte-endothelial adherence via A2B receptors [14].

Therefore, for the first time, we investigated the effects of fentanyl, sufentanil and remifentanil (as representative opioids) on monocyte-endothelial adherence, as well as the underlying mechanisms. We determined whether these opioids influence monocyte-endothelial adherence via ATP release mediated by Cx43 channels. We found that sufentanil, but not fentanyl or remifentanil, enhances monocyte-endothelial adherence via the inhibition of Cx43 channel function on monocytes. This side-effect of sufentanil should be considered seriously by clinicians.

## Methods

### Cell cultures

The study protocol conformed to the ethical guidelines of the 1975 Declaration of Helsinki and was approved by the Institutional Medical Ethics Committee of the Third Affiliated Hospital of Sun Yat-sen University.

The human umbilical vein endothelial cells (HUVECs) and U937 monocytes used in this study were both purchased from American Type Culture Collection (Manassas, VA, USA). HUVECs were cultured in human endothelial serum-free medium (Invitrogen, Carlsbad, CA, USA) with 20% fetal bovine serum (Invitrogen), 100 U/ml penicillin-streptomycin (Invitrogen), 100 μg/ml heparin (Sigma-Aldrich, St. Louis, MO, USA), and 150 μg/ml endothelial cell growth supplement (Becton, Dickinson and Company, Frankin Lakes, NJ, USA). U937 monocytes were cultured in RPMI1640 medium (Invitrogen), which contains 20% fetal bovine serum (Invitrogen) and 100 U/ml penicillin-streptomycin (Invitrogen). HUVECs and U937 monocytes were both cultured in an incubator (5% CO_2_, 37 °C, and 90% humidity) (Thermo Fisher Scientific, Waltham, MA, USA).

### Cell counting kit-8 assay

Cell vitality was detected using cell counting kit-8 kit assays (Dojindo Molecular Technologies, Inc., Kumamoto, Japan), according to the manufacturer’s instructions.

### Cell treatments

According to the requirements of each experiment, HUVECs and U937 monocytes were pre-treated with different chemicals, including 18-α-GA (a connexin channel inhibitor; 50 μM, for 1 h; Sigma-Aldrich) and Gap 27 (a connexin mimetic peptide that inhibits Cx43 channel function; 300 μM, for 1 h; Sigma-Aldrich); α, β-methylene ADP (APCP; a CD73 inhibitor; 300 μM, for 1 h; Sigma-Aldrich), exogenous ATP (200 μM, for 1 h; Sigma-Aldrich), and ADO (100 μM, for 1 h; Sigma-Aldrich). All the inhibitors, such as 18-α-GA, Gap 27 and APCP, including exogenous ATP and ADO were pre-treated for 1 h before cell adhesion assay and other experiments. Fentanyl (10 μg/ml, for 24 h; Yichang Humanwell Pharmaceutical Co., LTD, Yichang, Hubei, China), sufentanil (25 ng/ml, for 24 h; Yichang Humanwell Pharmaceutical Co., LTD), and remifentanil (50 ng/ml, for 24 h; Yichang Humanwell Pharmaceutical Co., LTD). All of the three anaesthetics were pre-treated for 24 h before cell adhesion assay and other experiments. (According to the available reports, we noticed that the concentrations of these anaesthetics were different significantly in vitro study. For example, the concentration of fentanyl was 0.01 μM − 150 μM (about 0.00336 μg/ml-50 μg/ml) [15–17]; the concentration of sufentanil was 0.5 ng/ml-50 ng/ml [18]; the concentration of remifentanil was 10 ng/ml-100 ng/ml [19, 20]. Supplementary Fig. 1 showed that fentanyl at the concentration of 50 μg/ml had cytotoxicity on HUVECs, so we chose 10 μg/ml in other experiments. Other concentrations of the three anaesthetics had no cytotoxicity on HUVECs or U937. Supplementary Fig. 2 showed that the different effects of fentanyl, remifentanil and sufentanil on cell adhesion. Fentanyl (0.1 μg/ml to 10 μg/ml) and remifentanil (0.05 ng/ml to 50 ng/ml) had no effects on cell adhesion when they acted on HUVECs or U937. Sufentanil (0.025 ng/ml to 25 ng/ml) had no influence on cell adhesion when it acted on HUVECs, but increased cell adhesion at the concentrations of 2.5 ng/ml to 25 ng/ml when it acted on U937. Therefore, we chose sufentanil at the concentration of 25 ng/ml in other experiments.)

### Adhesion assay

U937-HUVECs adhesion was detected according to procedures described previous studies: U937 monocytes were first labelled with calcein-acetoxymethyl ester (5 μM, Invitrogen) for 30 min in the incubator. The labelled U937 monocytes were then washed twice and resuspended in a serum-free medium. The labelled U937 monocytes were counted and poured onto confluent HUVEC monolayers, which had been pre-treated for 12 h with recombinant mouse tumour necrosis factor α (10 ng/mL; Peprotech, Rocky Hill, NJ, USA) before adhesion assay. Then, the plates were incubated for 1 h and rinsed twice slightly with the serum-free medium (All the inhibitors and anaesthetics were remained in the culture medium during the adhesion assay). Adherent U937 monocytes remained on HUVECs and were counted with a fluorescence microscope (Olympus IX71, Tokyo, Japan). Eight different 200× visual fields in each well were selected for analysis [3]. All the data was normalized to the control. Normalized data were used for statistics.

### ATP and ADO release detection

ATP release was detected with ATP bioluminescence assay kits (Sigma-Aldrich). The supernatants of HUVEC and U937 monocyte cultures were harvested on ice. One hundred microliters of supernatant were added to 100 μl of ATP assay mix solution in 96-well culture plates. The luminescence was read by a fluorospectrophotometer (Cary Eclipse, FL0811M005; bio/chemi-luminescence mode). The ADO content was detected using related ELISA kits (Xinyu Biotechnology, Shanghai, China), according to the manufacturer’s instructions [4].

#### Protein detection

Whole-cell lysates for western blotting were prepared by washing the cells twice with cell wash buffer [0.01 mol/l PBS, 0.138 mol/l NaCl, 0.02% NaN3 (pH 7.4)] followed by a 2 h incubation in lysis buffer (Nanjing Keygen Biotech Co., Ltd., Nanjing, China) at 4 °C. Proteins samples were quantified with Pierce™ BCA Protein assay kits (Thermo Fisher Scientific, Inc.). The protein sample (25 μg) was added into SDS-PAGE on 13% Tris-glycine mini-gels (Invitrogen Life Technologies) and transferred onto polyvinylidene difluoride membrane (Bio-Rad Laboratories, Inc., Hercules, CA, USA). After blocking with 5% milk for 1 h at room temperature, the membranes were incubated with Cx43 antibody overnight at 4 °C (anti-Cx43; 1:3000; Cat: SAB4501174, Sigma-Aldrich) and anti-β-tubulin for 1 h (1:10000; Cat: T4026, Sigma-Aldrich). Following several washes, the membranes were incubated for 1 h at room temperature with anti-mouse horseradish peroxidase (HRP)-conjugated secondary antibodies (1:3000, goat polyclonal antibody raised against mouse IgG; cat. no. M6898; Sigma-Aldrich). Protein band sizes were estimated with Alpha View software (version number: 2.2.14407, Protein Simple, Santa Clara, CA, USA). The original blots were showed in Supplementary Fig. 3.

### Parachute dye-coupling assay

Parachute dye-coupling assays were used to detect gap junction function in HUVECs. HUVECs were grown to confluence. The cells in one dish were as donor cells, which were labelled with calcein-AM (5 μM) in the incubator for 30 min. The calcein-AM went into the donor cells. They were green under a fluorescence microscope. After several washes with medium, donor cells were seeded onto receiver cells without calcein-AM. The donor: receiver cells ratio was 1: 150. After 4 h incubation, the results were observed with a fluorescence microscope (Olympus DP73, Tokyo, Japan). The green fluorescence would flow into the neighboring cells (receiver cells) through gap junction composed of connexins. The average number of receiver cells around every donor cell was counted; this reflected the function of Cx43 channels [21]. All the data was normalized to the control. Normalized data were used for statistics.

### Statistical analysis

Statistical analysis was performed using SPSS 15.0 software (SPSS, Inc., Chicago, IL, USA). All data are presented as mean ± S.D.. Multiple comparisons among groups were performed using repeated-measures one-way analyses of variance, followed by Tukey post hoc comparisons. All graphs were made by Sigmaplot 10.0 (Systat Software, Inc., Chicago, IL) and formed by the graph properties.

## Results

### Cx43 expressed on monocytes modulated U937-HUVECs adhesion via ATP release

**Fig. 1:**
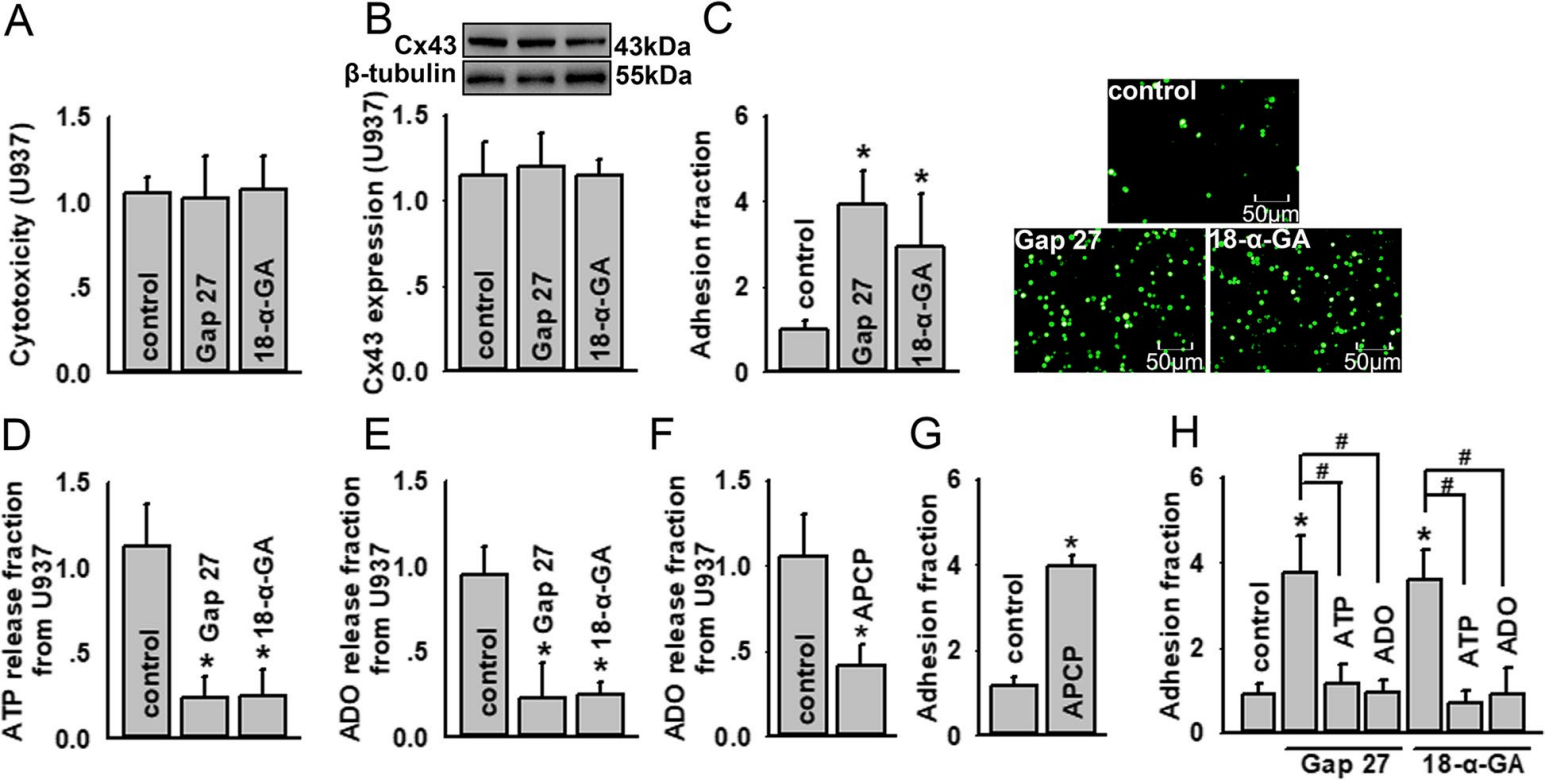
Cx43 expressed on monocytes modulates U937-HUVECs adhesion via ATP release. **a** Gap 27 and 18-α-GA induced no cytotoxicity in U937 monocytes (*n* = 5). Cell vitality is detected using cell counting kit-8 kit assays. The data of absorbance are normalized to control. **b** Gap 27 and 18-α-GA have no effects on Cx43 expression on U937 monocytes (*n* = 4). Cx43 expression is detected by western blotting. The gray values of blots are normalized to control. **c** U937-HUVECs adhesion is increased when U937 monocytes are pre-treated with Gap 27 and 18-α-GA (*n* = 4, *P < 0.05 vs control). U937-HUVECs adhesion is detected by adhesion assays. The data of adhesion fraction are normalized to control. **d** ATP release from U937 monocytes is reduced when U937 monocytes are pre-treated with Gap 27 and 18-α-GA (*n* = 6, *P < 0.05 vs control). ATP release is detected with ATP bioluminescence assay kits. The intensity of bioluminescence is normalized to control. **e** The ADO content is reduced when U937 monocytes are pre-treated with Gap 27 and 18-α-GA (*n* = 6, *P < 0.05 vs control). **f** The ADO content is reduced when U937 monocytes are pre-treated with APCP (*n* = 5, *P < 0.05 vs control). The ADO content is detected using related ELISA kits. The data of absorbance are normalized to control. **g** U937-HUVECs adhesion is increased when U937 monocytes are pre-treated with APCP (*n* = 5, *P < 0.05 vs control). **h** Application of exogenous ATP and ADO reduces U937-HUVECs adhesion increase provoked by 18-α-GA and Gap 27 (*n* = 5, *P < 0.05 vs control; # *P* < 0.05). U937-HUVECs adhesion is detected by parachute dye-coupling assays. The data of adhesion fraction are normalized to control. Gap 27: 300 μM, for 1 h; 18-α-GA: 50 μM, for 1 h; APCP: 300 μM, for 1 h; exogenous ATP: 200 μM, for 1 h; exogenous ADO: 100 μM, for 1 h. ADO, adenosine; APCP, α, β-methylene ADP; HUVEC, human umbilical vein endothelial cell. All experiments are conducted in the presence of TNF-α. All data are presented as mean ± S.D.. Multiple comparisons among groups are performed using repeated-measures one-way analyses of variance, followed by Tukey post hoc comparisons

As previously reported, 18-α-GA (a Cx43 channel inhibitor) and Gap 27 (a connexin mimetic peptide) effectively attenuated Cx43 channel function [4, 22]. As shown in Fig. 1a-c, when U937 monocytes were pre-treated with 18-α-GA or Gap 27 for 1 h, there was no effect on U937 survival or Cx43 expression (Fig. 1a, b); however, U937-HUVECs adhesion was increased significantly (Fig. 1c), indicating that inhibiting the function of Cx43 channels in U937 monocytes resulted in monocyte-endothelial adherence increase.

Cx43 channels are known to be permeable to ATP [23], and ATP can be rapidly metabolized to ADO by extracellular enzymes. Additionally, ADO has well-known anti-inflammatory effects that decrease monocyte-endothelial adherence by interacting with A2B receptors [24]. Therefore, we investigated the involvement of ATP release from U937 monocytes via Cx43 channels in the regulation of U937-HUVECs adhesion. As shown in Fig. 1d and e, the inhibition of Cx43 channels in U937 monocytes via 18-α-GA and Gap 27 administration attenuated ATP release from U937 monocytes, as well as the ADO content. Thus, important factors that resist monocyte-endothelial adherence were weakened.

Extracellular enzymes known to be involved in conversion of ATP to ADO are CD39 (converting ATP to AMP) and CD73 (converting AMP to ADO). This pathway is robust in endothelial cells. To confirm the function of ADO on U937-HUVEC adhesion, we used APCP (a competitive inhibitor of CD73) to inhibit the production of ADO from ATP [25]. APCP application on U937 monocytes resulted in the reduction of ADO, and subsequently caused a significant increase in U937-HUVEC adhesion (Fig. 1f, g). These results strongly suggested that ADO production from endogenously released ATP had a potent anti-adhesive effect. In order to confirm this conclusion, we supplied exogenous ATP and ADO to reverse the effects of 18-α-GA and Gap 27 on U937-HUVEC adhesion. As shown in Fig. 1h, the application of exogenous ATP and ADO reduced the increase of U937-HUVEC adhesion provoked by 18-α-GA and Gap 27 administration, demonstrating the effects of ATP and ADO on monocyte-endothelial adherence from another viewpoint.

### Cx43 expressed on HUVECs had no effect on U937-HUVEC adhesion

**Fig. 2:**
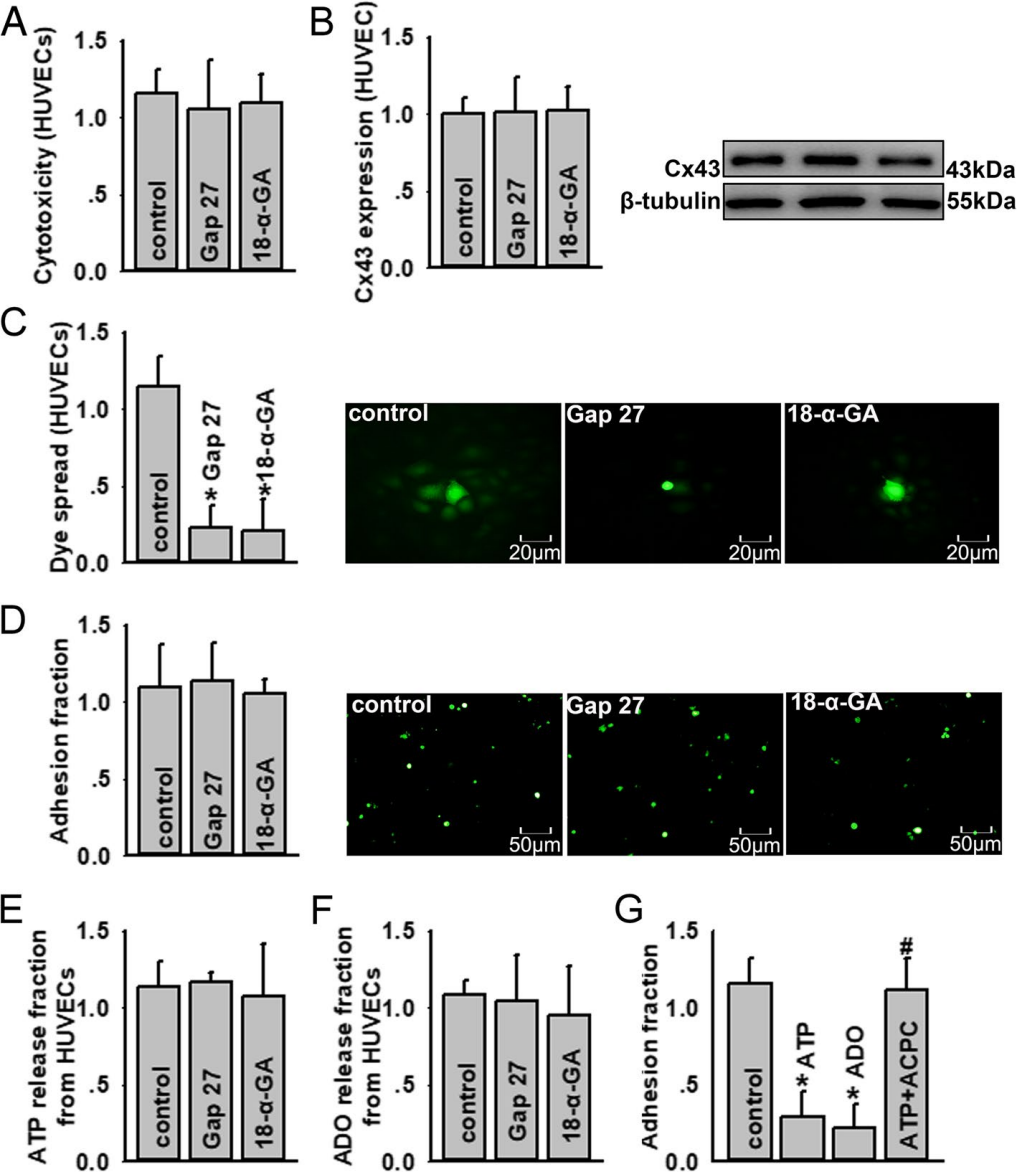
Cx43 expressed on HUVECs has no effects on U937-HUVECs adhesion. **a** Gap 27 and 18-α-GA have no cytotoxic effects on HUVECs (*n* = 4). Cell vitality is detected using cell counting kit-8 kit assays. The data of absorbance are normalized to control. **b** Gap 27 and 18-α-GA have no effects on Cx43 expression on HUVECs (*n* = 4). Cx43 expression is detected by western blotting. The gray values of blots are normalized to control. **c** Gap 27 and 18-α-GA inhibit dye coupling between HUVECs (*n* = 5, *P < 0.05 vs control). The dye spread between HUVECs is detected by dye-coupling assay. The number of receiver cells around donor cell are normalized to control. **d** Gap 27 and 18-α-GA have no effects on U937-HUVEC adhesion. U937-HUVECs adhesion is detected by adhesion assays. The data of adhesion fraction are normalized to control. **e** ATP release from HUVECs is not changed when HUVECs are pre-treated with Gap 27 and 18-α-GA (*n* = 5). ATP release is detected with ATP bioluminescence assay kits. The intensity of bioluminescence is normalized to control. **f** The ADO content is not changed when HUVECs are pre-treated with Gap 27 and 18-α-GA (*n* = 5). The ADO content is detected using related ELISA kits. The data of absorbance are normalized to control. **g** Exogenous application of ATP and ADO reduces U937-HUVEC adhesion (*n* = 5, *P < 0.05 vs control); APCP reverses U937-HUVEC adhesion decrease provoked by exogenous ATP (*n* = 5, *P < 0.05 vs control; #*P* < 0.05 vs ATP group). U937-HUVECs adhesion is detected by adhesion assays. The data of adhesion fraction are normalized to control. Gap 27: 300 μM, for 1 h; 18-α-GA: 50 μM, for 1 h; APCP: 300 μM, for 1 h; exogenous ATP: 200 μM, for 1 h; exogenous ADO: 100 μM, for 1 h. ADO, adenosine; APCP, α, β-methylene ADP; HUVEC, human umbilical vein endothelial cell. All experiments are conducted in the presence of TNF-α. All data are presented as mean ± S.D. Multiple comparisons among groups are performed using repeated-measures one-way analyses of variance, followed by Tukey post hoc comparisons

Cx43 is also expressed on HUVECs; therefore, we investigated the effects of Cx43 expressed on HUVECs on monocyte-endothelial adherence. As shown in Fig. 2a-c, 18-α-GA and Gap 27 pre-treatment on HUVECs had no influence on HUVEC survival or Cx43 expression, but they attenuated dye coupling between HUVECs, indicating that Cx43 channel function was reduced. Although 18-α-GA and Gap 27 pre-treatment inhibited Cx43 channel function in HUVECs, both agents had no effect on U937-HUVEC adhesion (Fig. 2d). We speculated that this contradictory phenomenon was because the ATP or ADO released from HUVECs was not changed (Fig. 2e, f and Supplementary Fig. 8). When we supplemented exogenous ATP and ADO, U937-HUVEC adhesion was decreased. The application of APCP on HUVECs caused an increase in U937-HUVEC adhesion, because APCP inhibited ADO production from ATP (Fig. 2g). These results suggest that Cx43 expressed on HUVECs did not modulate monocyte-endothelial adherence via ATP release.

### Effects of fentanyl, sufentanil, and remifentanil on U937-HUVEC adhesion

**Fig. 3:**
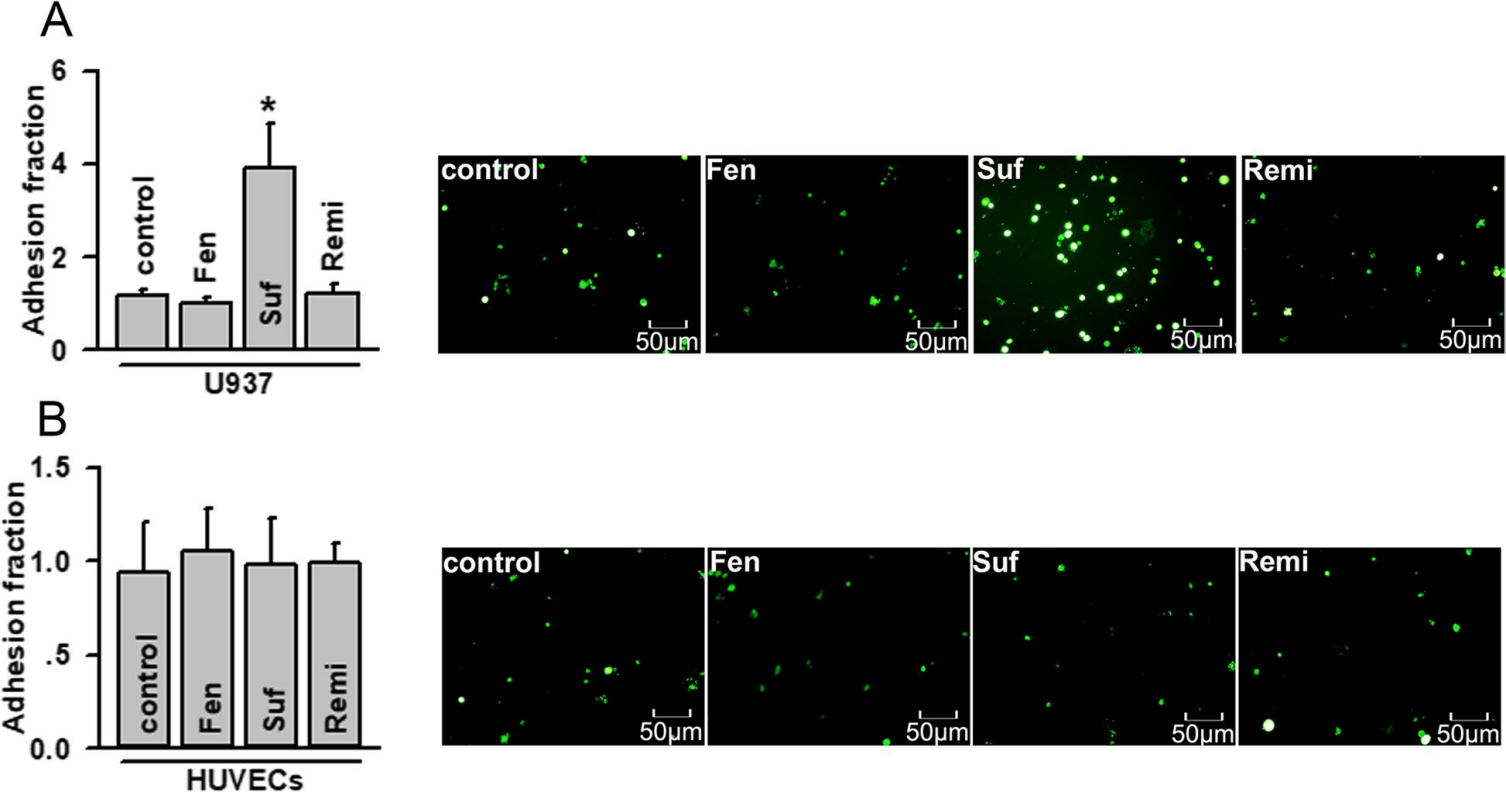
Effects of fentanyl, sufentanil, and remifentanil on U937-HUVEC adhesion. **a** Effects on U937-HUVEC adhesion when U937 monocytes are pre-treated with fentanyl, sufentanil, and remifentanil (*n* = 6, *P < 0.05 vs control). U937-HUVECs adhesion is detected by adhesion assays. The data of adhesion fraction are normalized to control. **b** Effects on U937-HUVEC adhesion when HUVECs are pre-treated with fentanyl, sufentanil, and remifentanil (*n* = 6, *P < 0.05 vs control). U937-HUVECs adhesion is detected by adhesion assays. The data of adhesion fraction are normalized to control. Fentanyl (Fen): 10 μg/ml, for 24 h; sufentanil (Suf): 25 ng/ml, for 24 h; remifentanil (Remi): 50 ng/ml, for 24 h. HUVEC, human umbilical vein endothelial cell. All experiments are conducted in the presence of TNF-α. All data are presented as mean ± S.D.. Multiple comparisons among groups are performed using repeated-measures one-way analyses of variance, followed by Tukey post hoc comparisons

Fentanyl, sufentanil, and remifentanil are commonly used in the clinic for patients with cancer or undergoing surgery; however, their effects on monocyte-endothelial adherence are unknown. We found that the effects of these opioids on monocyte-endothelial adherence differed, depending on whether they acted on monocytes or endothelial cells. When acting on U937 monocytes, sufentanil significantly increased U937-HUVEC adhesion; in contrast, fentanyl and remifentanil did not have this effect (Fig. 3a). However, when acting on HUVECs, none of these analgesics influenced U937-HUVEC adhesion (Fig. 3b).

### Sufentanil attenuated Cx43 channel function in HUVECs but had no effect on ATP or ADO release

**Fig. 4:**
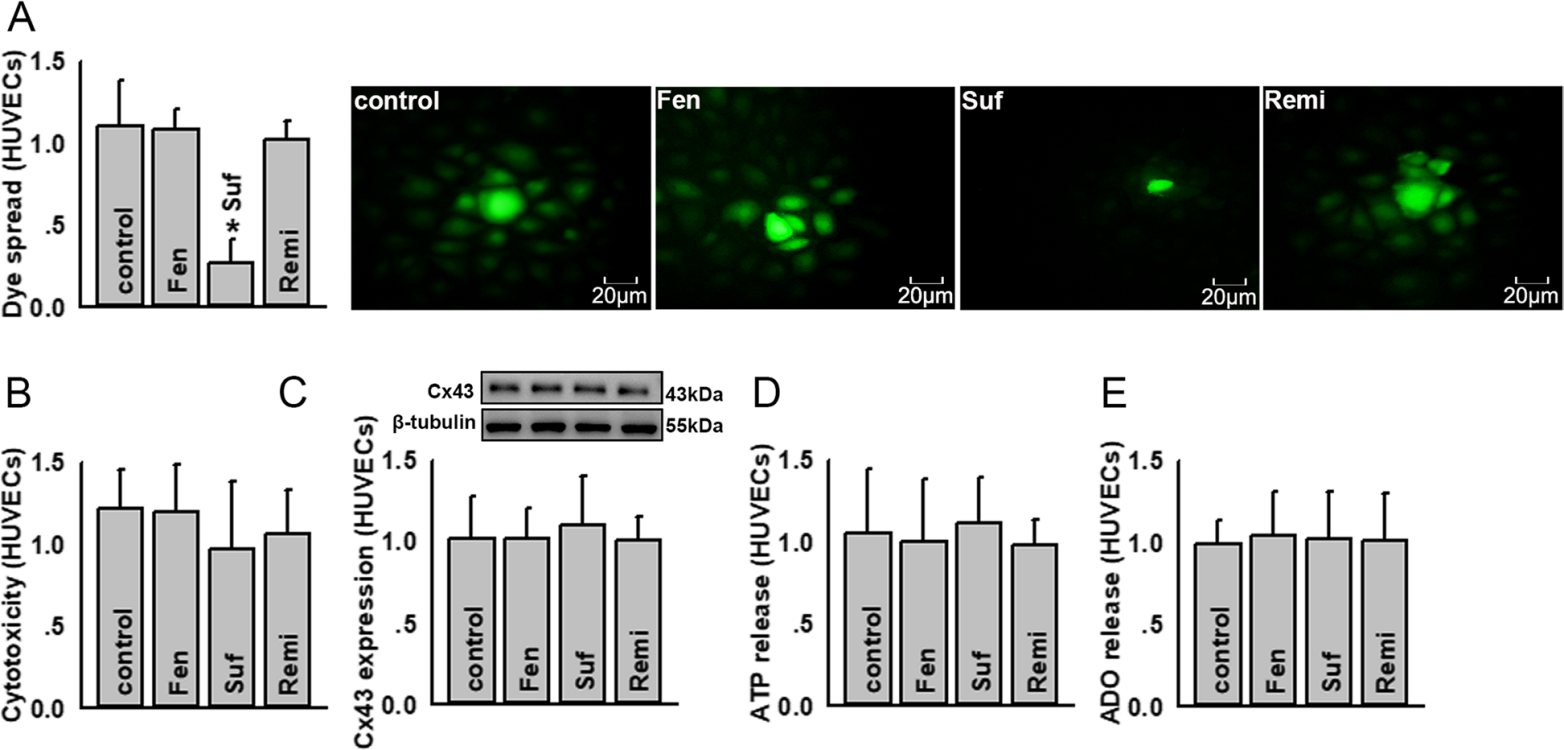
Sufentanil attenuates Cx43 channel function on HUVECs, with no effect on ATP and ADO release. **a** Sufentanil inhibits dye coupling between HUVECs, but fentanyl and remifentanil do not have this effect (*n* = 4, *P < 0.05 vs control). The dye spread between HUVECs is detected by dye-coupling assay. The number of receiver cells around donor cell are normalized to control. **b** Fentanyl, sufentanil, and remifentanil induced no cytotoxicity in HUVECs (*n* = 5). Cell vitality is detected using cell counting kit-8 kit assays. The data of absorbance are normalized to control. **c** Fentanyl, sufentanil and remifentanil have no effects on Cx43 expression in HUVECs (*n* = 5). Cx43 expression is detected by western blotting. The gray values of blots are normalized to control. **d** Fentanyl, sufentanil, and remifentanil have no effects on ATP release from HUVECs (*n* = 6). ATP release is detected with ATP bioluminescence assay kits. The intensity of bioluminescence is normalized to control. **e** Fentanyl, sufentanil, and remifentanil have no effects on the ADO content from HUVECs (*n* = 6). The ADO content is detected using related ELISA kits. The data of absorbance are normalized to control. Fentanyl (Fen): 10 μg/ml, for 24 h; sufentanil (Suf): 25 ng/ml, for 24 h; remifentanil (Remi): 50 ng/ml, for 24 h. ADO, adenosine; HUVEC, human umbilical vein endothelial cell. All experiments are conducted in the presence of TNF-α. All data are presented as mean ± S.D.. Multiple comparisons among groups are performed using repeated-measures one-way analyses of variance, followed by Tukey post hoc comparisons

U937-HUVECs adhesion is regulated by ATP release via Cx43 channels, as shown in Fig. 1; the effects of fentanyl, sufentanil, and remifentanil on ATP release from HUVECs are shown in Fig. 4. Although sufentanil inhibited Cx43 channel function in HUVECs (without affecting HUVEC survival or Cx43 expression) (Fig. 4a-c), it had no influence on ATP or ADO release from HUVECs (Fig. 4d, e and Supplementary Fig. 8). This might be the reason why sufentanil had no effect on U937-HUVECs adhesion when acting on HUVECs, even though it could inhibit Cx43 channel function on HUVECs. Fentanyl and remifentanil had no effects on Cx43 expression, Cx43 channel function in HUVECs, and ATP and ADO release (Fig. 4).

### Sufentanil, but not fentanyl or remifentanil, attenuated ATP and ADO release from U937 monocytes, affecting U937-HUVEC adhesion

**Fig. 5:**
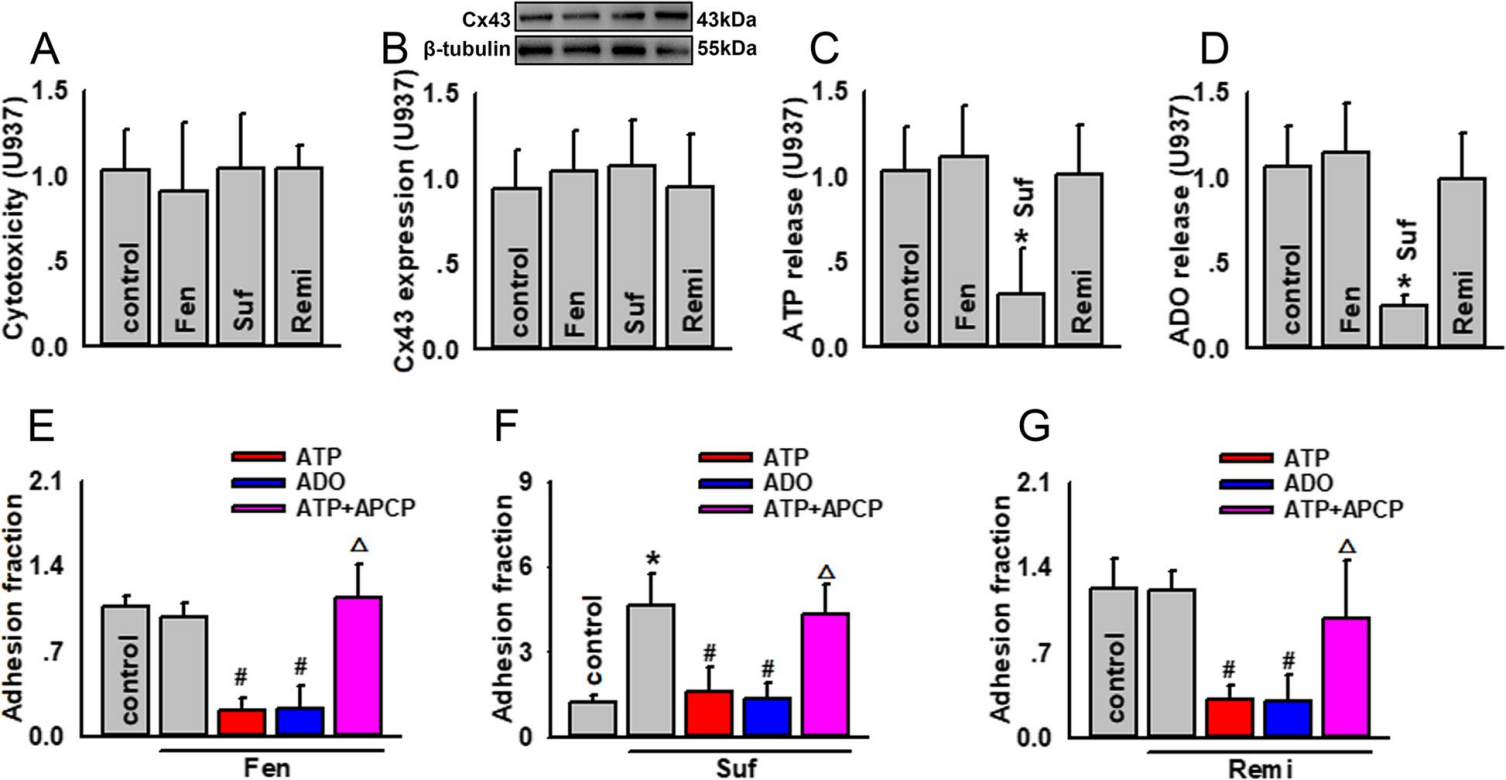
Sufentanil, but not fentanyl or remifentanil, attenuates ATP/ADO release from U937 monocytes, affecting U937-HUVECs adhesion. **a** Fentanyl, sufentanil, and remifentanil have no cytotoxic effects on U937 monocytes (*n* = 4). Cell vitality is detected using cell counting kit-8 kit assays. The data of absorbance are normalized to control. **b** Fentanyl, sufentanil, and remifentanil have no effects on Cx43 expression in U937 monocytes (*n* = 4). Cx43 expression is detected by western blotting. The gray values of blots are normalized to control. **c** Sufentanil, but not fentanyl or remifentanil, attenuates ATP release from U937 monocytes (*n* = 4, *P < 0.05 vs control). ATP release is detected with ATP bioluminescence assay kits. The intensity of bioluminescence is normalized to control. **d** Sufentanil, but not fentanyl or remifentanil, attenuates the ADO content from U937 monocytes (*n* = 4, *P < 0.05 vs control). The ADO content is detected using related ELISA kits. The data of absorbance are normalized to control. **e** Application of exogenous ATP and ADO reduces U937-HUVEC adhesion, when U937 monocytes are pre-treated with fentanyl; APCP reverses the decrease in U937-HUVEC adhesion provoked by exogenous ATP (#*P* < 0.05 vs fentanyl group; △*P* < 0.05 vs fentanyl+ATP group); **f** Application of exogenous ATP and ADO reduces U937-HUVEC adhesion, when U937 monocytes are pre-treated with sufentanil; APCP reverses the decrease in U937-HUVEC adhesion provoked by exogenous ATP (*n* = 5, *P < 0.05 vs control; #*P* < 0.05 vs fentanyl group; △*P* < 0.05 vs sufentanil+ATP group); **g** Exogenous ATP and ADO reduce U937-HUVEC adhesion, when U937 monocytes are pre-treated with remifentanil; APCP reverses the decrease in U937-HUVEC adhesion provoked by exogenous ATP (#*P* < 0.05 vs fentanyl group; △*P* < 0.05 vs remifentanil +ATP group). U937-HUVECs adhesion is detected by adhesion assays. The data of adhesion fraction are normalized to control. Fentanyl (Fen): 10 μg/ml, for 24 h; sufentanil (Suf): 25 ng/ml, for 24 h; remifentanil (Remi): 50 ng/ml, for 24 h; APCP: 300 μM, for 1 h; exogenous ATP: 200 μM, for 1 h; exogenous ADO: 100 μM, for 1 h. ADO, adenosine; APCP, α, β-methylene ADP; HUVEC, human umbilical vein endothelial cell. All experiments are conducted in the presence of TNF-α. All data are presented as mean ± S.D.. Multiple comparisons among groups are performed using repeated-measures one-way analyses of variance, followed by Tukey post hoc comparisons

Figure 5a and b show that fentanyl, sufentanil, and remifentanil had no effects on U937 survival and Cx43 expression; however, sufentanil application obviously attenuated ATP and ADO release from U937 monocytes, while fentanyl and remifentanil did not show these effects (Fig. 5c, d).

The pre-treatment of U937 monocytes with exogenous ATP or ADO reversed the increase in U937-HUVEC adhesion induced by treatment with sufentanil (fentanyl and remifentanil themselves did not affect U937-HUVEC adhesion, but exogenous ATP and ADO also attenuated U937-HUVEC adhesion). When APCP was used to inhibit ADO production from ATP, the anti-adhesion effect of ATP disappeared (Fig. 5e-g), suggesting that ATP converting to ADO played an important part in the anti-adhesion effect.

Supplementary Fig. 4a-c showed that with Cx43 over-expression on U937 monocytes, ATP and ADO release from U937 monocytes were increased significantly, but U937-HUVEC adhesion was also increased (Supplementary Fig. 4d). This phenomenon contradicted our results in Fig. 1 that ATP and ADO could reduce U937-HUVEC adhesion. Our previous reports had demonstrated that Cx43 over-expression on U937 monocytes increased U937-HUVEC adhesion via modulating PKC-α/NOX2/ROS signaling pathway [26]. Therefore, we speculated that the function of ATP reducing U937-HUVEC adhesion was reversed by Cx43 over-expression. Our results in this part also supported this speculation. With the application of sufentanil, ATP and ADO release from U937 monocytes were also depressed, and conversely, U937-HUVEC adhesion increased furtherly (Supplementary Fig. 4).

## Discussion

Opioids have been used for many years in clinical practice, especially for cancer pain management, anaesthesia, and postoperative analgesia [27, 28]. Studies on the side effects of opioids have mainly focused on nausea, vomiting, postoperative delirium, and breathing effects, and the effects of opioids on monocyte-endothelial adherence are unknown [29]. For the first time, we investigated the effects of representative opioids, including fentanyl, sufentanil, and remifentanil, on monocyte-endothelial adherence. The present study showed that when these three opioids acted on monocytes or endothelial cells, their effects on monocyte-endothelial adherence differed. When acting on U937 monocytes, sufentanil significantly increased U937-HUVEC adhesion which was associated with reduced release of ATP from Cx43 channels. In contrast, fentanyl and remifentanil had no influence on U937-HUVECs adhesion or ATP release. Although sufentanil could also inhibit Cx43 channel function in HUVECs, it had no effect on ATP release from HUVECs, as well as U937-HUVEC adhesion, further indicating that ATP release might be the main reason Cx43 channels can regulate U937-HUVECs adhesion.

In Supplementary Fig. 5, we studied the effect of sufentanil on U937-HUVEC adhesion under different physiological and therapeutic conditions. The results showed that at the condition of HUVECs untreated with TNF-α, U937-HUVEC adhesion was not changed, no matter U937 monocytes or HUVECs were pretreated with sufentanil (Supplementary Fig. 5a). In contrast, at the condition of HUVECs treated with TNF-α, sufentanil increased adhesion fraction when U937 monocytes were pretreated with sufentanil, but had no effects on adhesion fraction when HUVECs were pretreated with sufentanil (Supplementary Fig. 5b), which was coincident with our results in Fig. 3. When U937 monocytes and HUVECs were both pretreated with sufentanil at the same time, adhesion fraction was also increased and the extent of increase was just the same as that U937 monocytes were pretreated with sufentanil (Supplementary Fig. 5b). These results in Supplementary Fig. 5 demonstrated that sufentanil only acted under inflammatory conditions.

Under normal physiological conditions, the interaction of flowing monocytes with vascular endothelial cells is minimal. However, circulating monocytes are prone to adherence to vascular endothelial cells under pathological conditions, especially when the vascular endothelial cells are inflamed and damaged [30, 31]. There are numerous risk factors, such as surgical stimulation, inflammation and vascular injury induced by surgery, long durations in the supine position, and chemotherapies, that could result in monocyte-endothelial adherence in patients with cancers or undergoing surgery [32–34]. However, the effects of analgesics on monocyte-endothelial adherence have not been previously reported. Fentanyl, sufentanil and remifentanil are all widely used for different kinds of pain control, not only for surgical patients, but also for patients with cancer. Their side-effects on monocyte-endothelial adherence should be considered by clinicians.

Fentanyl, sufentanil and remifentanil selectively target the μ-opioid receptor, which belongs to the G protein-coupled receptor family [35]. Supplementary Fig. 7 showed that the 3 main classes of opioid receptors, mu (MOR), kappa (KOR), and delta (DOR) were expressed on both HUVECs and U937 monocytes. Conformational flexibility is one of the most essential characteristics of G protein-coupled receptors, which are involved in ligand recognition and subsequent activation or inactivation [36]. Compared to the other two opioids, sufentanil is the agonist with the highest affinity toward μ-opioid receptors. It has been reported that sufentanil could regulate the activity of G protein via μ-receptors, and with the knockdown of G protein by siRNA, Cx43 channel function was inhibited [37, 38]. This may explain why sufentanil can interact with μ-opioid receptors and activate the downstream signalling pathways of G proteins, but fentanyl and remifentanil cannot. In order to confirm the effects of μ-opioid receptors on U937-HUVECs adhesion, we used the selective antagonist of μ-opioid receptors, β-funaltrexamine, to pretreat U937 monocytes in Supplementary Fig. 9. The results showed that β-funaltrexamine effectively reversed the influence of sufentanil on ATP release and U937-HUVECs adhesion, increasing ATP release from U937 monocytes (Supplementary Fig. 9A) and attenuating U937-HUVECs adhesion (Supplementary Fig. 9B), which demonstrated that μ-opioid receptors also played an important part in sufentanil increasing monocyte-endothelial adherence. According to the available reports, we notice that Cx43 channel function could be regulated by G protein. As a result of inhibition with pertussis toxin (PTX; a specific inhibitor of certain G proteins), the number of plasma membrane hemichannels available for Cx43 channels assembly is reduced [39]. Another report demonstrates that GTP-binding protein-coupled receptor (GPCR) agonist alters the interaction between Cx43 and its molecular partners, regulating Cx43 channel function [40]. This might also be the reason why sufentanil could alter Cx43 channel function, while fentanyl and remifentanil did not have this kind of effect. From another aspect, Supplementary Fig. 2c showed that sufentanil resulted in monocyte-endothelial adherence increase in a concentration-dependent manner. Therefore, we speculate that sufentanil application may influence the activity of G protein via acting on μ-opioid receptor, resulting in the inhibition of Cx43 channels.

The reports about effects of sufentanil on vascular inflammatory diseases are very limited. In our present study, we demonstrated that sufentanil application could increase monocyte-endothelial adherence, which initiated or deteriorated vascular inflammatory diseases. Nevertheless, previous study indicated that sufentanil inhibited migration of human leukocytes through human endothelial cell monolayers, reducing the occurrence of vascular inflammatory diseases, but its possible mechanism had not been clarified [18]. It means that sufentinal plays various roles in different stages of vascular inflammatory diseases. We speculate that the different influences on monocyte-endothelial adherence and cells migration are still related with the changes of Cx43 channel function. As the close of Cx43 channels by sufentanil, ATP release from monocytes is reduced, resulting in monocyte-endothelial adherence increase. Simultaneously, the calcium current is also inhibited. It has been widely accepted that calcium is one of the few substances that could be efficiently transferred through the Cx43 channels, and it provides power for cell movement [41–44]. Therefore, when Cx43 channels are inhibited by sufentanil, calcium current is cut off, leading to cells migration inhibited. It further indicates that Cx43 plays various roles in sufentanil addecting vascular inflammatory diseases.

The current study also confirmed our previous conclusion [4]: depending on whether Cx43 channel function in U937 monocytes or HUVECs was altered, its effects on ATP release were different. When Cx43 channel function in U937 monocytes was inhibited, ATP release was attenuated; in contrast, altering function of Cx43 channels expressed on HUVECs did not affect ATP release. U937 monocytes are a kind of suspend cells, Cx43 in plasma membranes of U937 monocytes is prone to form unopposed hemichannels, which mediate substance exchange inside and outside cells. Therefore, ATP or ADO could be easily released to the outside cells, inhibiting cell adhesion [45]. In contrast, the neighboring HUVECs are closely linked. Cx43 hemichannels exist on the neighboring HUVECs dock together to form integral channels, mainly mediating small molecular, such as ATP, movement between neighboring HUVECs, but not make them released to the outside cells [46]. That might be the reason why the inhibitors, 18-α-GA and Gap 27, inhibited Cx43 channel function, but had no effect on ATP release in HUVECs (Fig. 2a-d).

The mechanism of ATP release mediated by Cx43 hemichannels has been well clarified. ATP is rapidly metabolized to ADO by extracellular enzymes; ADO has well-known anti-inflammatory effects that decrease monocyte-endothelial adherence through interaction with A2B receptors. The enzymes involved are CD39, which converts ATP to AMP, and CD73, which converts AMP to ADO. From the available evidence, this pathway, which protects against cell adhesion, is robust in endothelial cells [47, 48]. As previously reported, in addition to Cx43 channels, both connexin37 (Cx37) channels and pannexin1 are potential pathways for ATP release. These two proteins are both expressed in U937 monocytes and HUVECs (Supplementary Fig. 6). However, among all connexin channels, homomeric Cx37 channels are the most size-restrictive in terms of being impermeable to Lucifer yellow, Alexa 488, and 6-carboxyfluorescein and are only weakly permeable to some smaller molecules, such as NBD-MTMA and Alexa 350 [49, 50]. Therefore, we favour the idea that ATP release from monocytes is via channels formed wholly and/or partially by Cx43 (homomeric Cx43 and/or heteromeric Cx43/Cx37 channels). All available facts indicate that Cx43 channels expressed on monocytes play an important role in monocyte-endothelial adherence. The other potential pathway relevant to ATP release involves pannexin1, but 18-α-GA at a very low concentration, as used in the current study, has no effects on pannexin1 [51]. The analyses all suggest that the release of ATP from monocytes is rapidly and dynamically regulated by Cx43 channel function.

Although the present results demonstrated that sufentanil application might increase U937-HUVEC adhesion under inflammatory conditions, which was associated with reduced release of ATP from Cx43 channels, all the experiments were carried out in vitro. We know that the situation in vivo is much more complicated than that in vitro and in vitro experiments have limited support for clinical views, but these results at least remind us that sufentanil might have a new side-effect that it exacerbates monocyte adhesion to endothelial cells, which is worth studying in vivo or using freshly isolated primary cells in future.

## Conclusions

With the development of society, the consumption of opioids has tended to increase worldwide [52]. The present results demonstrate that sufentanil application might increase U937-HUVEC adhesion which was associated with reduced release of ATP from Cx43 channels. In contrast, fentanyl and remifentanil do not show these effects. The side-effects of opioids on monocyte-endothelial adherence should be seriously taken into consideration by clinicians.

## Supplementary Information

**Additional file 1.**

## Data Availability

The datasets used and/or analysed during the current study are available from the corresponding author on reasonable request.

## Abbreviations

ADO: Adenosine
APCP: α, β-methylene ADP
Cx37: Connexin37
Cx43: Connexin43
HUVEC: Human umbilical vein endothelial cell
