# Supplementary material for: A new side-effect of sufentanil: increased monocyte-endothelial adhesion
Source: BMC Anesthesiol. 2021 Nov 3;21:267. doi: 10.1186/s12871-021-01487-3 (PMC8565079; doi:10.1186/s12871-021-01487-3)
Supplement: Supplementary file 1 — Additional file 1. [file 12871_2021_1487_MOESM1_ESM.pdf]

A new side-effect of sufentanil: increased monocyte-endothelial adhesion

Dongdong Yuan[1#]*, Zhaowei Zou[2#], Xianlong Li[1], Nan Cheng[1], Na Guo[1], Guoliang Sun[1]*, Dezhao Liu[1]*

[1]Department of Anesthesiology, the third affiliated hospital of Sun Yat-sen university, Tianhe Road, Guangzhou, Guangdong, P. R. China.

[2]Department of General Surgery, Zhujiang Hospital, Southern Medical University, Guangzhou, China

#These authors contributed equally to this study.

***Correspondence:**

Dezhao Liu, Guoliang Sun, and Dongdong Yuan

Department of Anesthesiology, the Third affiliated hospital of Sun Yat-sen University, Tianhe Road, Guangzhou, Guangdong, P. R. China. Tel: +86-20-85253132, Email: sumsldz@126.com; yuandongdong123@126.com

Methods

1. Cx43 over-expression

Cx43 was over-expressed in U937 monocytes with a pcDNA3.1- Cx43 vector (gift of Ryan Jensen and Peter M. Glazer) [1]. Transfection into U937 monocytes was carried out using Lipofectamine 2000 according to the manufacturer's instructions (Invitrogen). After 72 h, western blotting was used to assess Cx43 expression.

2. Real-time polymerase chain reaction (RT-PCR) [2, 3]

Total RNA extracted from HUVECs and U937 monocytes using Trizol reagent (Thermo Fisher Scientific, Waltham, MA, USA). We used the NanoDrop-1000 spectrophotometer (Thermo Fisher Scientific) to detect RNA quality and concentration. Reverse transcription was performed using ReverTra Ace qPCR RT Master Mix (Toyobo, Osaka, Japan). Quantitative analysis was conducted with quantitative RT-PCR (qRT-PCR) using SYBR® Green Realtime PCR Master Mix (Toyobo) with Roche LightCycler 1.1. The sense and antisense oligonucleotide primers were as follows: MOR, (f) TACCGTGTGCTATGGACTGAT, (r) ATGATGACGTAAATGTGAATG; DOR, (f) GCGGGAAAGCCAGTGACTC (r) TGCCCTGTTTAAGGACTCAGTTG; KOR, (f) CGTCTGCTACACCCTGATGATC, (r) CTCTCGGGAGCCAGAAAGG. Glyceraldehyde 3-phosphate dehydrogenase (GAPDH) was used as the housekeeping gene, (f) GGAAGCTCACTGGCATGGC(r)TAGACGGCAGGTCAGGTCCA. Data of transcripts were calculated relative to GAPDH using the $2^{-\Delta\Delta Ct}$ method.

Supplementary results

1. The cytotoxicity of fentanyl, sufentanil and remifentanil on HUVECs and U937, and effects of these three anaesthetics on cell adhesion at different concentrations.

According to the available reports, we noticed that the concentrations of these anaesthetics were different significantly in vitro study. For example, the concentration of fentanyl was 0.01 μM -150 μM (about 0.00336 μg/ml-50 μg/ml) [4-6]; the concentration of sufentanil was 0.5 ng/ml-50 ng/ml [7]; the concentration of remifentanil was 10 ng/ml-100 ng/ml [8, 9]. Supplementary Figure 1 showed that fentanyl at the concentration of 50 μg/ml had cytotoxicity on HUVECs, so we chose 10 μg/ml in other experiments. Other concentrations of the three anaesthetics had no cytotoxicity on HUVECs or U937. Supplementary Figure 2 showed that the different effects of fentanyl, remifentanil and sufentanil on cell adhesion. Fentanyl (0.1 μg/ml to 10 μg/ml) and remifentanil (0.05 ng/ml to 50 ng/ml) had no effects on cell adhesion when they acted on HUVECs or U937. Sufentanil (0.025 ng/ml to 25 ng/ml) had no influence on cell adhesion when it acted on HUVECs, but increased cell adhesion at the concentrations of 2.5 ng/ml to 25 ng/ml when it acted on U937. Therefore, we chose sufentanil at the concentration of 25 ng/ml in other experiments.

Supplementary Figure 1

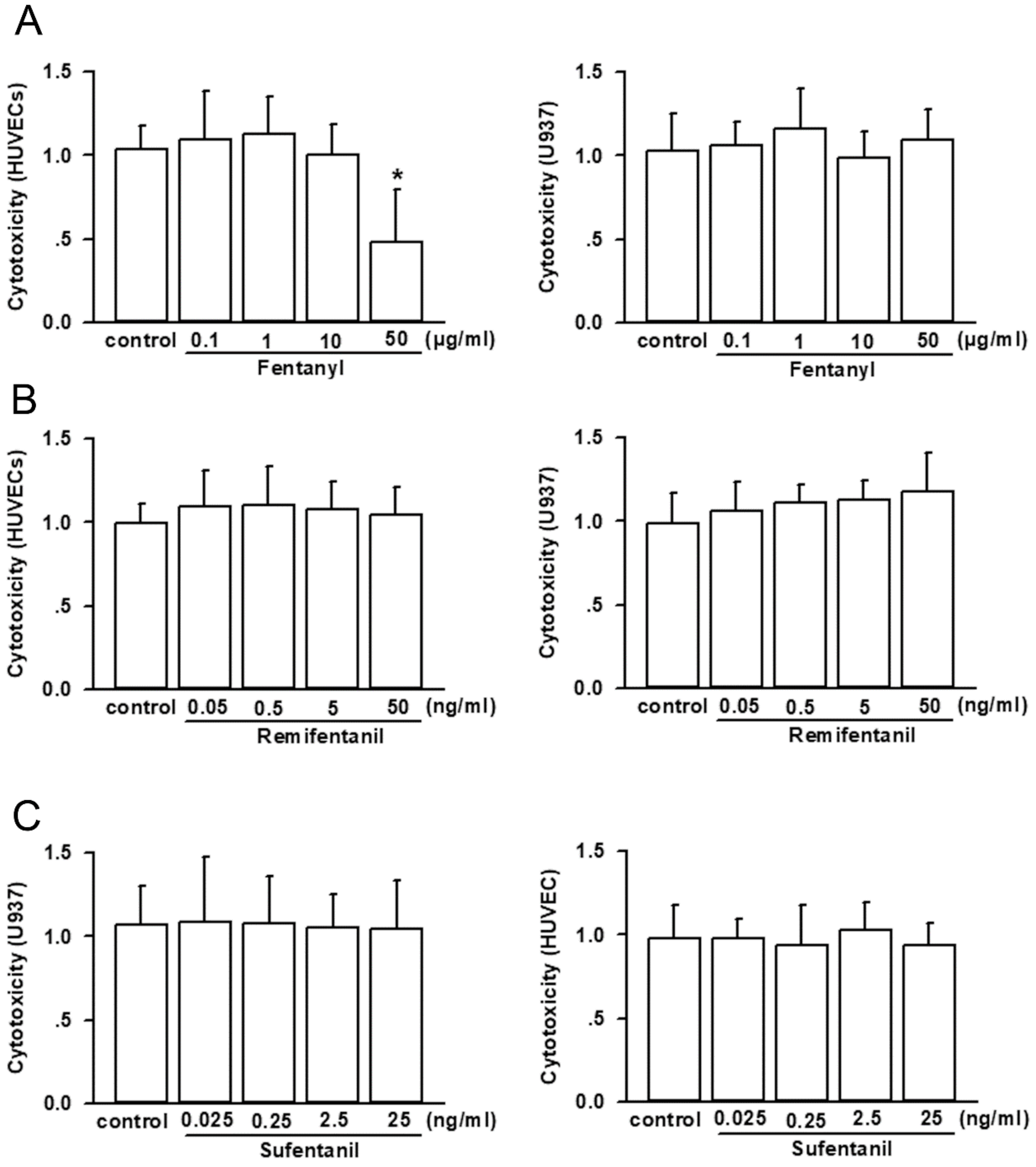

Supplementary Figure 1. The cytotoxicity of fentanyl, sufentanil and remifentanil on HUVECs and U937 when they acted on HUVECs or U937 at different concentrations for 24 hours. (a) The cytotoxicity of fentanyl at 0.01 μM -150 μM (about 0.00336 μg/ml-50 μg/ml) on HUVECs and U937. n = 4, *P<0.05 vs control; (b) The cytotoxicity of remifentanil at 10 ng/ml-100 ng/ml on HUVECs and U937. n = 4; (c) The cytotoxicity of sufentanil at 0.5 ng/ml-50 ng/ml on HUVECs and U937. n = 4. Cell

vitality is detected using cell counting kit-8 kit assays. The data of absorbance are normalized to control. All experiments are conducted in the presence of TNF-α. All data are presented as mean ± S.D.. Multiple comparisons among groups are performed using repeated-measures one-way analyses of variance, followed by Tukey post hoc comparisons.

Supplementary Figure 2

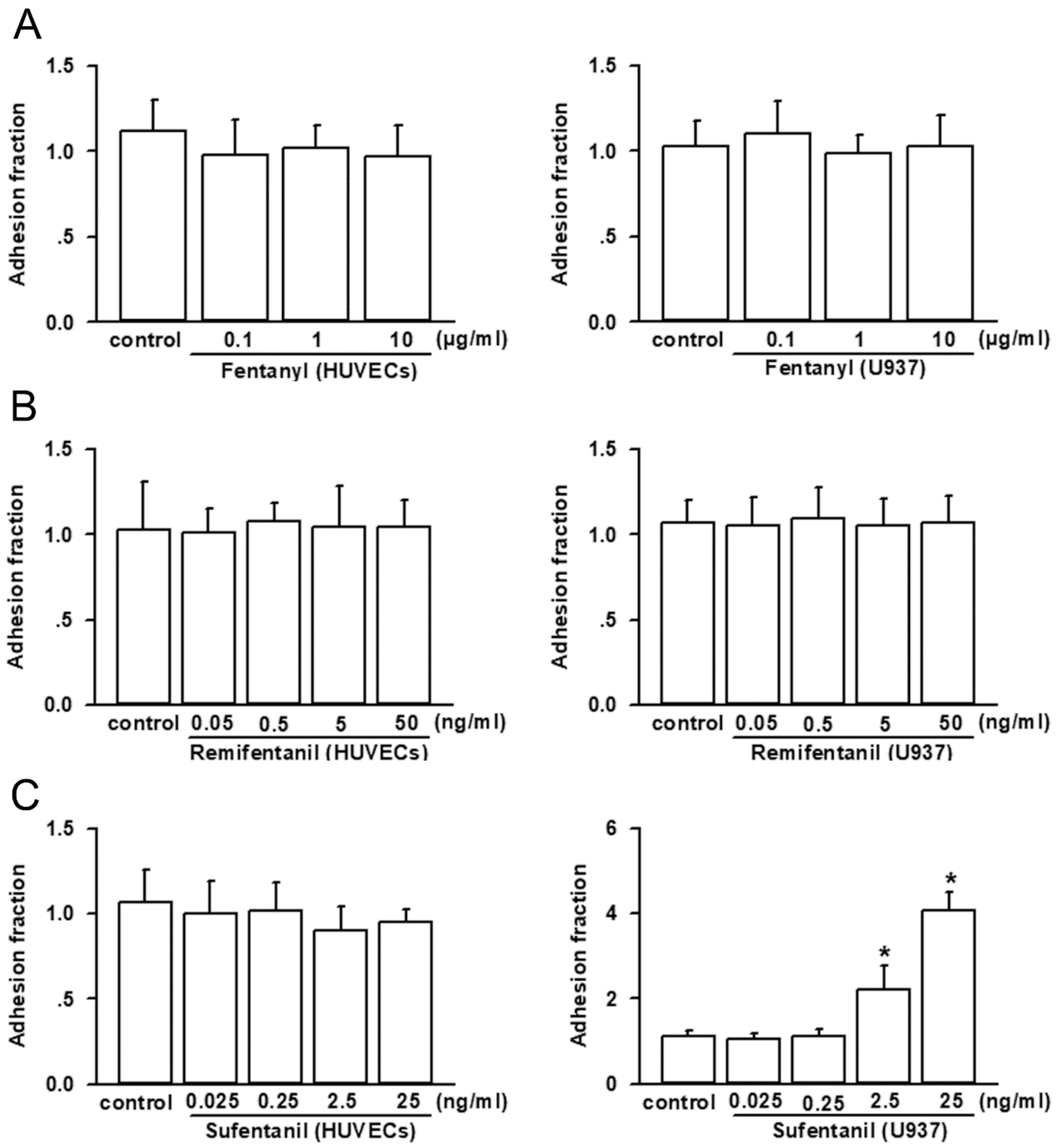

Supplementary Figure 2. The effects of fentanyl, sufentanil and remifentanil on cell adhesion when they acted on HUVECs or U937 at different concentrations for 24 hours. (a) The effects of fentanyl on cell adhesion at 0.1 μg/ml-10 μg/ml, when they acted on HUVECs or U937 for 24 hours. n = 3; (b) The effects of remifentanil on cell adhesion at 0.05 ng/ml-50 ng/ml, when they acted on HUVECs or U937 for 24 hours. n = 3; (c) The effects of sufentanil on cell adhesion at 0.025 ng/ml-25 ng/ml, when they acted on HUVECs or U937 for 24 hours. n = 3, *P<0.05 vs control. U937-HUVECs adhesion is detected by adhesion assays. The data of adhesion fraction are normalized to control. All experiments are conducted in the presence of TNF-α. All data are presented as mean ± S.D.. Multiple comparisons among groups are performed using repeated-measures one-way analyses of variance, followed by Tukey post hoc comparisons.

2. The original blots of western blotting

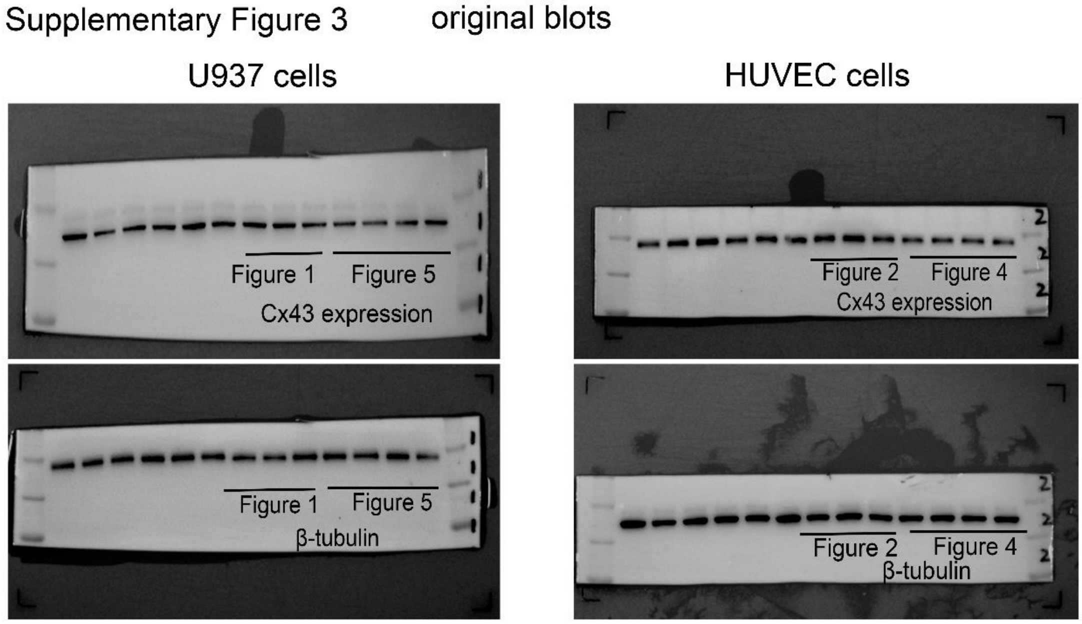

Supplementary Figure 3. The original blots of western blotting.

3. Effects of sufentanil on ATP and ADO release, and U937-HUVEC adhesion when Cx43 was over-expressed on U937 monocytes.

Supplementary Figure 4a-c showed that with Cx43 over-expression on U937 monocytes, ATP and ADO release from U937 monocytes were increased significantly, but U937-HUVEC adhesion was increased (Supplementary Figure 4d). This phenomenon contradicted our results in Figure 1 that ATP and ADO could reduce U937-HUVEC adhesion. Our previous reports had demonstrated that Cx43 over-expression on U937 monocytes increased U937-HUVEC adhesion via modulating PKC-α/NOX2/ROS signaling pathway [10]. Therefore, we speculated that the function of ATP reducing U937-HUVEC adhesion was reversed by Cx43 over-expression. Our results in this part also supported this speculation. With the application of sufentanil, ATP and ADO release from U937 monocytes were also depressed, and conversely, U937-HUVEC adhesion increased furtherly (Supplementary Figure 4).

Supplementary Figure 4

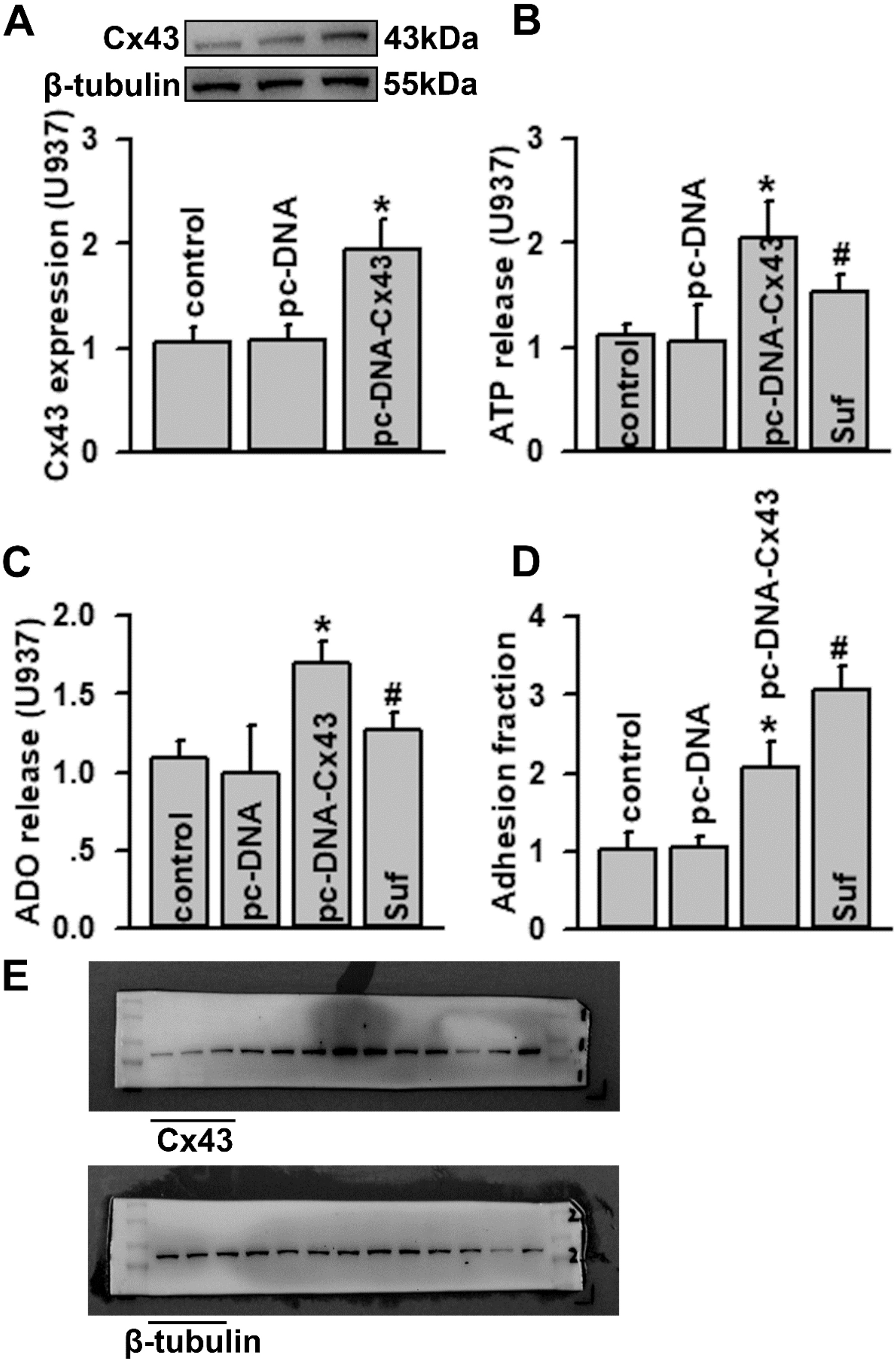

Original blots of Supplementary Figure 4A

Supplementary Figure 4. Effects of sufentanil on ATP and ADO release, and U937-

HUVEC adhesion when Cx43 is over-expressed on U937 monocytes. (a) Cx43 is over-expressed on U937 monocytes with pc-DNA-Cx43. (n=4, *$P<0.05$ *vs* control). Cx43 expression is detected by western blotting. The gray values of blots are normalized to control. (b) ATP release from U937 monocytes is increased with Cx43 over-expression, which could be reduced by sufentanil (n=3, *$P<0.05$ *vs* control; #$P<0.05$ *vs* pc-DNA-Cx43). ATP release is detected with ATP bioluminescence assay kits. The intensity of bioluminescence is normalized to control. (c) ADO release from U937 monocytes is also increased with Cx43 over-expression, which could be reduced by sufentanil (n=3, *$P<0.05$ *vs* control; #$P<0.05$ *vs* pc-DNA-Cx43). The ADO content is detected using related ELISA kits. The data of absorbance are normalized to control. (d) U937-HUVEC adhesion is also increased with Cx43 over-expression, which could be reduced by sufentanil (n=3, *$P<0.05$ *vs* control; #$P<0.05$ *vs* pc-DNA-Cx43). (e) Original bolts of Supplementary Figure 4(a). U937-HUVECs adhesion is detected by adhesion assays. sufentanil (Suf): 25 ng/ml, for 24 hours. The data of adhesion fraction are normalized to control. All experiments are conducted in the presence of TNF-α. All data are presented as mean ± S.D.. Multiple comparisons among groups are performed using repeated-measures one-way analyses of variance, followed by Tukey post hoc comparisons.

4. ***Effects of sufentanil on U937-HUVEC adhesion*** **when** ***HUVECs were pretreated with or without TNF-α.***

In Supplementary Figure 5, we studied the effect of sufentanil on U937-HUVEC adhesion under different physiological and therapeutic conditions. The results showed that at the condition of HUVECs untreated with TNF-α, U937-HUVEC adhesion was not changed, no matter U937 monocytes or HUVECs were pretreated with sufentanil (Supplementary Figure 5a). In contrast, at the condition of HUVECs treated with TNF-α, sufentanil increased adhesion fraction when U937 monocytes were pretreated with sufentanil, but had no effects on adhesion fraction when HUVECs were pretreated with sufentanil (Supplementary Figure 5b), which was coincident with our results in Figure 3. When U937 monocytes and HUVECs were both pretreated with sufentanil at the same time, adhesion fraction was also increased and the extent of increase was just the same as that U937 monocytes were pretreated with sufentanil (Supplementary Figure 5b).

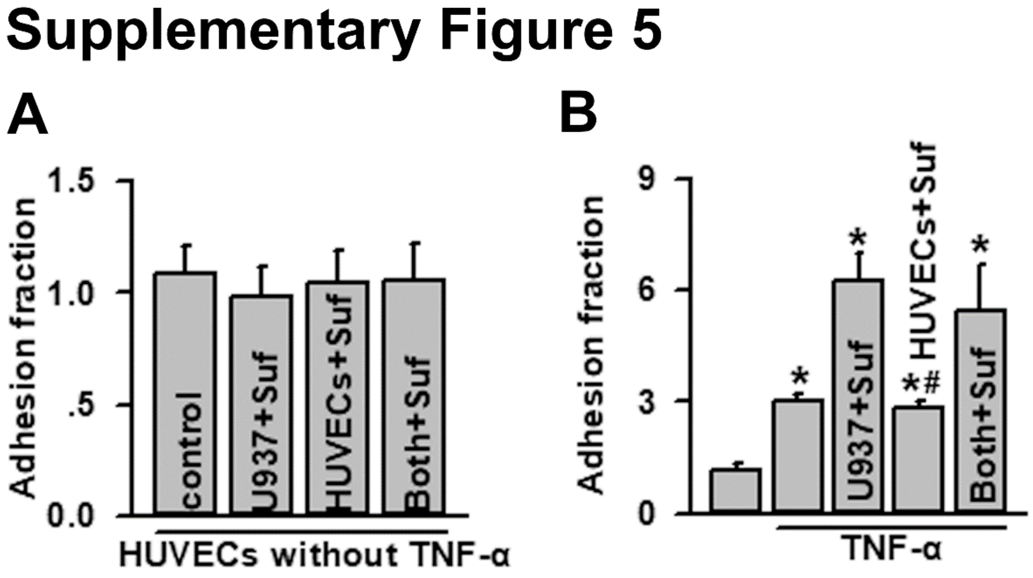

Supplementary Figure 5. Effects of sufentanil on U937-HUVEC adhesion when HUVECs were pretreated with or without TNF-α (n=3). (a) At the condition of

HUVECs untreated with TNF-α, effects of sufentanil on U937-HUVEC adhesion. (b) At the condition of HUVECs treated with TNF-α, effects of sufentanil on U937-HUVEC adhesion. (n=3, *P<0.05 *vs* control; #P<0.05 *vs* U937+Suf). Sufentanil (Suf): 25 ng/ml, for 24 hours. U937-HUVECs adhesion is detected by adhesion assays. The data of adhesion fraction are normalized to control. All data are presented as mean ± S.D.. Multiple comparisons among groups were performed using repeated-measures one-way analyses of variance, followed by Tukey post hoc comparisons.

5. Cx37 and pannexin-1 are expressed on both U937 monocytes and HUVECs.

According to different reports, we notice that there is mainly pannexin-1 expressed in monocytes, which mediates ATP release [11, 12]. Meanwhile, Cx37 channels are also considered to play an important role in regulating monocyte-endothelial adherence via regulating ATP release [13]. Therefore, we test pannexin-1 and Cx37 expression on U937 monocytes or HUVECs in this part. The results showed that Cx37 and pannexin-1 were expressed on both U937 monocytes and HUVECs.

Supplementary Figure 6

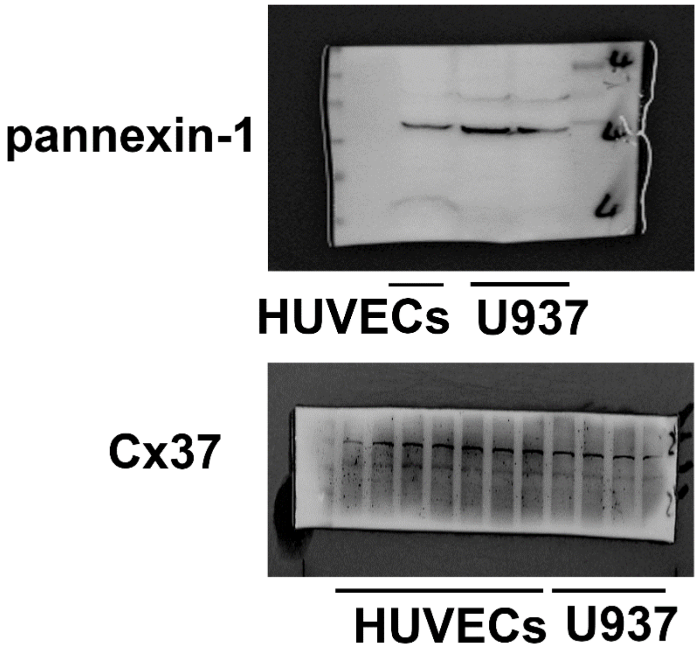

Supplementary Figure 6. Cx37 and pannexin-1 are expressed on both U937 monocytes and HUVECs. Both Cx37 and pannexin-1 expression are detected by western blotting.

6. Three main classes of opioid receptors, mu (MOR), kappa (KOR), and delta (DOR), were expressed on HUVECs and U937 monocytes and HUVECs.

We detected the 3 main classes of opioid receptors, mu (MOR), kappa (KOR), and delta (DOR) by qPCR. The results showed that all of the 3 opioid receptors were expressed on both HUVECs and U937 monocytes (Supplementary Figure 7).

Supplementary Figure 7

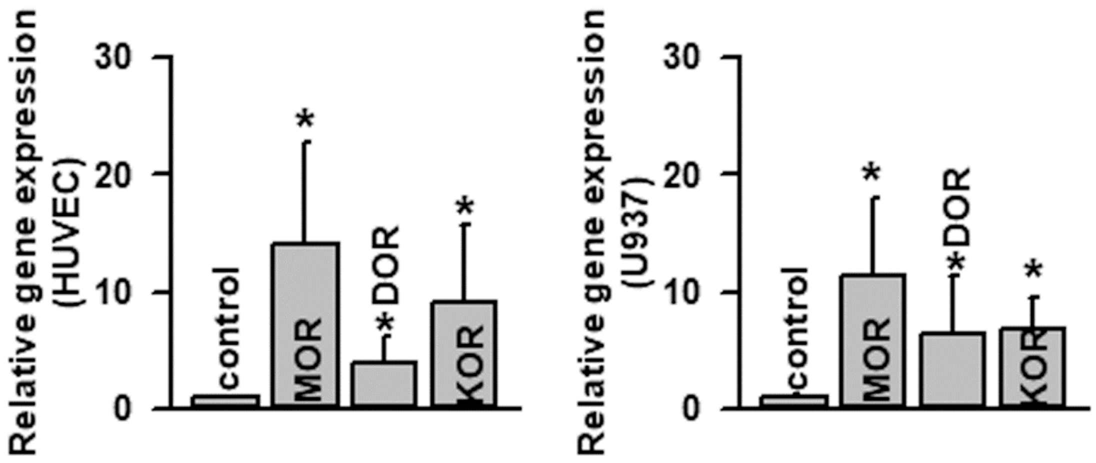

Supplementary Figure 7. Three main classes of opioid receptors, mu (MOR), kappa (KOR), and delta (DOR), were expressed on HUVECs and U937 monocytes and HUVECs (n=3, **P*<0.05 *vs* control). The control was blank control group without samples. The mRNAs of the 3 opioid receptors are detected by qPCR. All data are presented as mean ± S.D.. Multiple comparisons among groups were performed using repeated-measures one-way analyses of variance, followed by Tukey post hoc comparisons.

7. ATP release from HUVECs (the absolute ATP values).

We showed the absolute ATP values of HUVECs in Supplementary Figure 8. All of the chemicals used in this investigation, such as Gap 27, 18-α-GA, Fentanyl, Sufentanil and Remifentanil, had no effects on ATP release from HUVECs.

Supplementary Figure 8

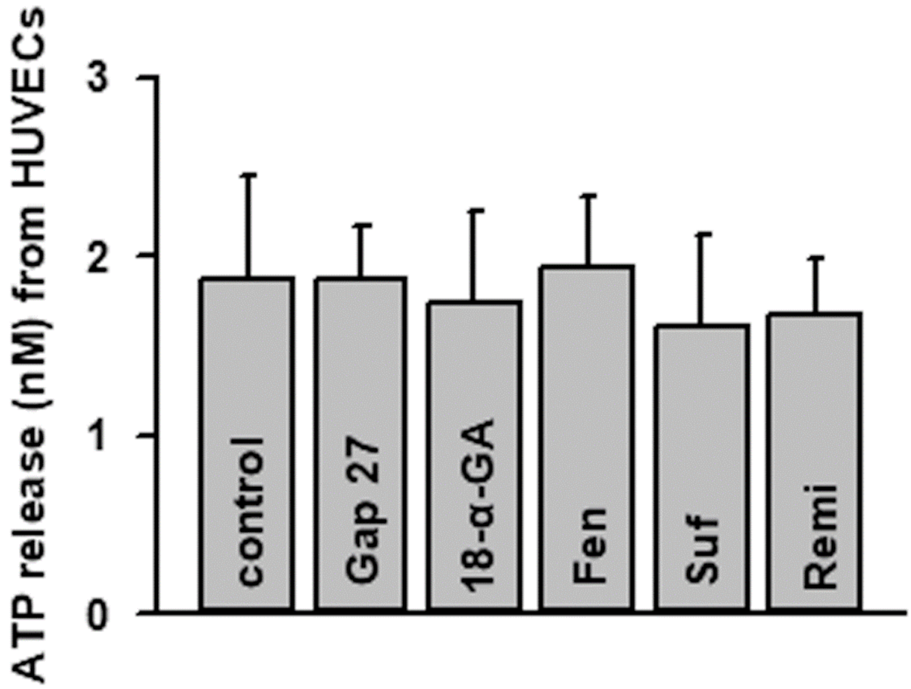

Supplementary Figure 8. ATP release from HUVECs (the absolute ATP values) when pretreated with Gap 27, 18-α-GA, Fentanyl, Sufentanil and Remifentanil (n=5). ATP release is detected with ATP bioluminescence assay kits. Gap 27: 300 µM, for 1 hour; 18-α-GA: 50 µM, for 1 hour; Fentanyl (Fen): 10 µg/ml, for 24 hours; sufentanil (Suf): 25 ng/ml, for 24 hours; remifentanil (Remi): 50 ng/ml, for 24 hours. HUVEC, human umbilical vein endothelial cell. All experiments are conducted in the presence of TNF-α. All data are presented as mean ± S.D.. Multiple comparisons among groups are performed using repeated-measures one-way analyses of variance, followed by Tukey post hoc comparisons.

8. The selective antagonist of µ-opioid receptors, β-funaltrexamine, reversed effects of sufentanil on ATP release from U937 monocytes and U937-HUVECs

adhesion.

In order to confirm the effects of μ-opioid receptors on U937-HUVECs adhesion, we used the selective antagonist of μ-opioid receptors, β-funaltrexamine, to pretreat U937 monocytes in Supplementary Figure 9. The results showed that β-funaltrexamine effectively reversed the influence of sufentanil on ATP release and U937-HUVECs adhesion, increasing ATP release from U937 monocytes (Supplementary Figure 9A) and attenuating U937-HUVECs adhesion (Supplementary Figure 9B), which demonstrated that μ-opioid receptors did play an important part in sufentanil increasing monocyte-endothelial adherence.

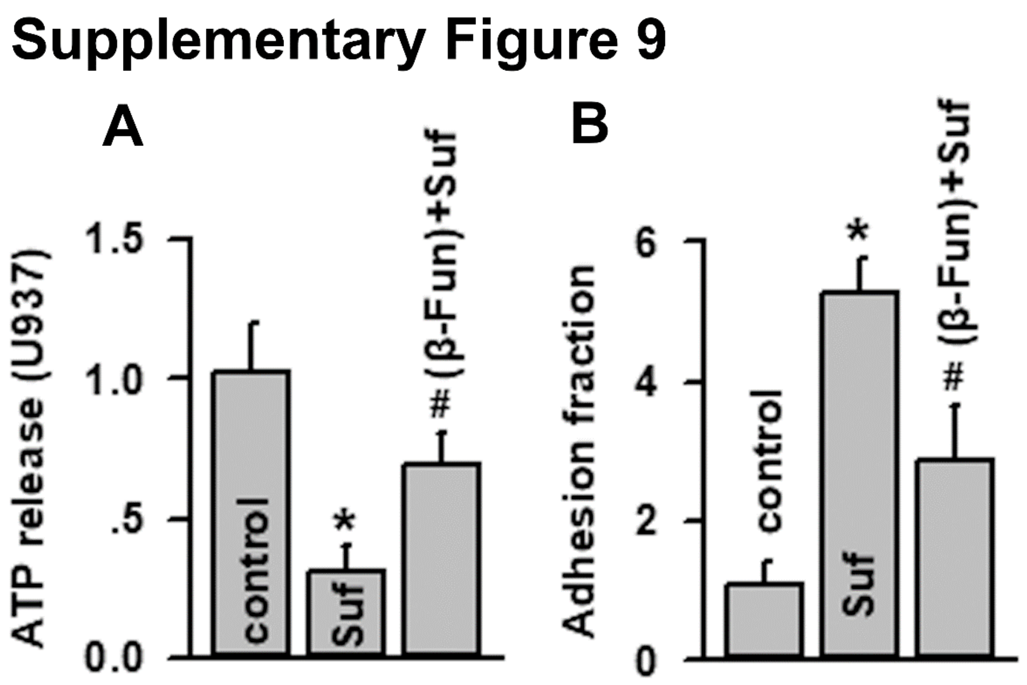

Supplementary Figure 9. The selective antagonist of μ-opioid receptors, β-funaltrexamine, reversed effects of sufentanil on ATP release from U937 monocytes and U937-HUVECs adhesion. (a) β-funaltrexamine increased ATP release from U937 monocytes (n=3, **P*<0.05 *vs* control; #*P*<0.05 *vs* Suf group). ATP release is detected

with ATP bioluminescence assay kits. (b) β-funaltrexamine attenuated U937-HUVECs adhesion (n=3, *P<0.05 *vs* control; #P<0.05 *vs* Suf group). U937-HUVECs adhesion is detected by adhesion assays. sufentanil (Suf): 25 ng/ml, for 24 hours; β-funaltrexamine (β-fun): 30μM, before sufentanil treatment for 1 hour (during sufentanil treatment for 24 hours, β-funaltrexamine still exists in the culture medium). The data of adhesion fraction are normalized to control. All experiments are conducted in the presence of TNF-α. All data are presented as mean ± S.D.. Multiple comparisons among groups are performed using repeated-measures one-way analyses of variance, followed by Tukey post hoc comparisons.

Supplementary Figure legends

1. Ji H, Qiu R, Gao X, Zhang R, Li X, Hei Z, Yuan D: **Propofol attenuates monocyte-endothelial adhesion via modulating connexin43 expression in monocytes**. *Life sciences* 2019, **232**:116624.
2. Chen CJ, Liu DZ, Yao WF, Gu Y, Huang F, Hei ZQ, Li X: **Identification of key genes and pathways associated with neuropathic pain in uninjured dorsal root ganglion by using bioinformatic analysis**. *Journal of pain research* 2017, **10**:2665-2674.
3. Peng J, Sarkar S, Chang SL: **Opioid receptor expression in human brain and peripheral tissues using absolute quantitative real-time RT-PCR**. *Drug Alcohol Depend* 2012, **124**(3):223-228.
4. Yao J, Ma C, Gao W, Liang J, Liu C, Yang H, Yan Q, Wen Q: **Fentanyl induces autophagy via activation of the ROS/MAPK pathway and reduces the sensitivity of cisplatin in lung cancer cells**. *Oncology reports* 2016, **36**(6):3363-3370.
5. Atcheson R, Rowbotham DJ, Lambert DG: **Fentanyl inhibits the release of [3H]noradrenaline from SH-SY5Y human neuroblastoma cells**. *British journal of anaesthesia* 1994, **72**(1):98-103.
6. Davis RL, Buck DJ, Saffarian N, Stevens CW: **The opioid antagonist, beta-funaltrexamine, inhibits chemokine expression in human astroglial cells**. *Journal of neuroimmunology* 2007, **186**(1-2):141-149.
7. Hofbauer R, Moser D, Salfinger H, Frass M, Kapiotis S: **Sufentanil inhibits migration of human leukocytes through human endothelial cell monolayers**. *Anesthesia and analgesia* 1998, **87**(5):1181-1185.
8. Yang Y, Chen C, Cui C, Jiao Y, Li P, Zhu L, Yu W, Xia Q, Wen D, Yang L: **Indispensable role**

of beta-arrestin2 in the protection of remifentanil preconditioning against hepatic ischemic reperfusion injury. *Scientific reports* 2019, **9**(1):2087.

9. Liu X, Yang H, Liu Y, Jiao Y, Yang L, Wang X, Yu W, Su D, Tian J: **Remifentanil upregulates hepatic IL-18 binding protein (IL-18BP) expression through transcriptional control**. *Laboratory investigation; a journal of technical methods and pathology* 2018, **98**(12):1588-1599.
10. Chai Y, Cao Z, Yu R, Liu Y, Yuan D, Lei L: **Dexmedetomidine Attenuates LPS-Induced Monocyte-Endothelial Adherence via Inhibiting Cx43/PKC-alpha/NOX2/ROS Signaling Pathway in Monocytes**. *Oxidative medicine and cellular longevity* 2020, **2020**:2930463.
11. Cymer M, Brzezniakiewicz-Janus K, Bujko K, Thapa A, Ratajczak J, Anusz K, Tracz M, Jackowska-Tracz A, Ratajczak MZ, Adamiak M: **Pannexin-1 channel "fuels" by releasing ATP from bone marrow cells a state of sterile inflammation required for optimal mobilization and homing of hematopoietic stem cells**. *Purinergic Signal* 2020, **16**(3):313-325.
12. Lee AH, Ledderose C, Li X, Slubowski CJ, Sueyoshi K, Staudenmaier L, Bao Y, Zhang J, Junger WG: **Adenosine Triphosphate Release is Required for Toll-Like Receptor-Induced Monocyte/Macrophage Activation, Inflammasome Signaling, Interleukin-1beta Production, and the Host Immune Response to Infection**. *Critical care medicine* 2018, **46**(12):e1183-e1189.
13. Wong CW, Christen T, Roth I, Chadjichristos CE, Derouette JP, Foglia BF, Chanson M, Goodenough DA, Kwak BR: **Connexin37 protects against atherosclerosis by regulating monocyte adhesion**. *Nature medicine* 2006, **12**(8):950-954.
